# The Use of 3D Printing in Fetal Surgery for Surgical Planning: A Scoping Review

**DOI:** 10.3390/jcm13174999

**Published:** 2024-08-23

**Authors:** Aaron J. Fils, Julia Kasmirski, Oluwateniayo Okpaise, John M. Reynolds, Gabriele Tonni, Heron Werner, Rodrigo Ruano

**Affiliations:** 1 Miller School of Medicine, University of Miami, Miami, FL 33163, USA; ajf179@med.miami.edu; 2 University of São Paulo Medical School, São Paulo 01246-903, Brazil; julia.kasmirski@gmail.com; 3 Medway Maritime Hospital, Gillingham ME7 5NY, UK; teniayookpaise@gmail.com; 4 Louis Calder Memorial Library, University of Miami Miller School of Medicine, Miami, FL 33136, USA; 5 Department of Obstetris & Neonatology, Istituto di Ricovero e Cura a Carattere Scientifico (IRCCS), ASL di Reggio Emilia, 42100 Reggio Emilia, Italy; gabriele.tonni@ausl.re.it; 6 Pontificia Universidade Catolica do Rio de Janeiro, Rio de Janeiro 22451-900, Brazil; heron.werner@gmail.com; 7 Division of Maternal-Fetal Medicine, Department of Obstetrics, Gynecology & Reproductive Sciences, University of Miami Miller School of Medicine, 1120 NW 14th Street, Suite # 1152, Miami, FL 33136, USA

**Keywords:** anatomical models, congenital abnormalities, diagnostic imaging, 3D printing, surgical planning, fetal surgery

## Abstract

**Objectives**: We sought to identify in which clinical scenarios 3D printed models are used to plan for fetal surgeries as well as the main purpose and the imaging method utilized for the models. In addition, we describe benefits and shortcomings of the models, as well as potential future improvements. **Methods**: In this scoping review, data were collected retrospectively from scientific databases (PubMed, Embase, Cochrane CENTRAL, CINAHL, Scopus, and the Web of Science platform) and screened by title, abstract, and full text against strict criteria. The inclusion criteria required the study be performed on a live fetus and involve 3D models used for fetal surgery. The models must have been designed from diagnostic imaging modalities such as CT, MRI, or ultrasound. The articles considered include clinical trials, review articles, cohort studies, case series, case reports, and conference abstracts. **Results**: Of the initial 742 articles collected, six met the inclusion criteria. Spina bifida and EXIT procedures were the most frequent use cases that inspired surgeons to print models for surgical planning. The ability to view patient-specific anatomy in a 3D handheld model was often touted as providing a great benefit to the surgical team’s ability to anticipate intraoperative challenges. **Conclusions**: Three-dimensional printing models have been applied to plan for fetal surgeries, more specifically, for EXIT procedures and fetoscopic surgical repair of spina bifida. The potential benefits of 3D printing in fetal surgery are enormous.

## 1. Introduction

Three-dimensional (3D) printing, invented by Charles Hull in the 1980s [1], enables the construction of physical 3D models. Though initially hindered by slow speed and limited materials, 3D printing has become a viable tool across numerous industries, including medicine.

The first step in creating a physical model is generating a digital model using computer-aided design (CAD) software, computed tomography (CT), magnetic resonance imaging (MRI), or ultrasound. Then, the digital model is optimized and exported for 3D printing. Lastly, the print material and printer settings are selected [2,3]. Generally, as speed increases, print quality will decrease.

Fetal medicine and surgery focus on diagnosing and treating congenital abnormalities during pregnancy. Sir William Liley’s in utero fetal transfusion for erythroblastosis fetalis in the 1960s marked the first therapeutic intervention on a fetus [4]. Early surgical attempts often resulted in fetal demise, due in part to the lack of effective tocolytic therapies and the high risk of preterm labor. Some current applications of fetal surgery include the correction of congenital diaphragmatic hernia, myelomeningocele, and complications of twin pregnancies [5]. Although surgical techniques have made remarkable progress, they have not been perfected, and they require substantial resources and dedicated training investments [6].

Three-dimensional printing also serves as an important adjunct to pre-procedural planning. Repetitive simulated practice allows a surgical team to finalize their surgical approach, anticipate intraoperative challenges, and optimize surgical instrumentation [7]. Steen et al.’s. [8] simulator for in utero gastroschisis repair adequately assessed the surgical skills required [8]. After a teaching session at the 2018 EuroCMR/SCMR joint congress, participants reported that visualizing complex cases can aid decision making. They unanimously agreed that implementing 3D printing in their practice is valuable [9]. Clearly, there is interest in this technology and the belief it will enhance surgical planning and performance. The integration of 3D printing imparts significant potential to advance the practice of fetal surgery and improve patient outcomes.

The present scoping review aims to comprehensively explore the utilization of 3D printing in fetal surgery planning. Unlike systematic reviews, which focus on specific empirical evidence to answer narrow research questions, scoping reviews are more flexible, allowing for a comprehensive review of the breadth of the literature in underexplored areas. The decision to conduct a scoping review, as opposed to other forms of research synthesis, was driven by the appropriateness of this method for mapping an area of research, rather than evaluating the impact of interventions on specific outcomes, as typically seen in systematic reviews. By examining the creation of digital 3D models of fetal anomalies and the techniques employed to print them as physical models, this review seeks to shed light on the diverse applications of 3D printing in fetal surgical care. The integration of 3D printing technology into this cutting-edge field holds significant potential to advance the practice of fetal surgery and improve patient outcomes.

## 2. Materials and Methods

We performed a scoping review of the use of 3D printing for surgical planning in fetal surgery, i.e., planning the type and mode of fetal procedure for a specific anomaly. The scoping review was conducted with guidance from the JBI Manual for Evidence Synthesis [10] and reported in accordance with Preferred Reporting Items for Systematic Reviews and Meta-Analyses Extension for Scoping Reviews (PRISMA-ScR) [11]. Scoping reviews represent a robust systematic method to provide an extensive overview of the current state of knowledge for a particular topic or field [12]. The study protocol was registered at Open Science Framework, https://doi.org/10.17605/OSF.IO/BEAGZ (accessed on 28 July 2024). 

### 2.1. Search Strategy

The search strategy was developed by the researcher (A.F.) and an academic health science librarian (J.R.). The search strategy was written for PubMed and translated using each database’s syntax, controlled vocabulary, and search fields. MeSH, EMTREE, and CINAHL subject headings and text words were used for the concepts of fetal surgery, three-dimensional additive printing, and their synonyms. We searched PubMed Medline (United States National Library of Medicine), EMBASE (Elsevier, Embase.com (accessed on 28 July 2024)), Cochrane CENTRAL (Cochrane Library, Wiley), CINAHL Plus with Full Text (Ebsco), SCOPUS (Elsevier), and the Web of Science platform (Clarivate: Science Citation Index Expanded, Social Sciences Citation Index, Arts & Humanities Citation Index, Conference Proceedings Citation Index-Science, Conference Proceedings Citation Index-Social Science & Humanities, Emerging Sources Citation Index, KCI-Korean Journal Database, ProQuest Dissertations & Theses Citation Index, SciELO Citation Index). All databases were searched on 12 August 2023. For full search strategies, see Appendix A. All database records were downloaded to EndNote 20 [13] and uploaded to Covidence web-based software for deduplication, screening, and full-text evaluation [14]. We did not contact any study authors, manufacturers, other experts, or search study registries. We reviewed the citations from the studies that met our inclusion criteria and any systematic or scoping reviews found using our search strategy. The Retraction Watch database was queried using Endnote software to ensure that no retracted studies were included.

### 2.2. Study/Source of Evidence Selection

All identified citations were collated and uploaded into the citation system Endnote and then into the review software Covidence. Uploaded citations were reviewed and duplicates were removed. Titles and abstracts were screened by two independent reviewers (J.K., A.F., or O.O.) to ensure that they followed the inclusion and exclusion criteria. The full text of selected citations was assessed in detail against the inclusion criteria by two or more independent reviewers (J.K., A.F. and O.O.). Reasons for the exclusion of sources of evidence at full text were recorded and reported in the scoping review. Any disagreements that arose between the reviewers at each stage of the selection process were resolved by a third reviewer and any further disputes were resolved through discussion. The results of the search and the study inclusion process were reported in full in the final scoping review and presented in a Preferred Reporting Items for Systematic Reviews and Meta-analyses extension for scoping review (PRISMA-ScR) flow diagram.

### 2.3. Data Extraction

Data were extracted from papers included in this scoping review in Covidence by three independent reviewers (J.K., A.F. and O.O.). The extracted data included specific details about the gestational age, mother’s age, fetal pathology, imaging methodology used to develop the 3D models, type of 3D printing employed (printing modality), 3D printing material used, 3D models of the fetal organ or fetal anomaly, type of fetal surgery performed, the utility of the 3D model, and the contribution of the 3D model to define the delivery mode when EXIT was considered.

The draft data extraction tool was revised as necessary during the process of extracting data from each included evidence source. Modifications were detailed in the scoping review. In the case of a disagreement between the two review authors, the article was placed in the conflict section to be further reviewed by a third author (J.K., A.F., or O.O.).

## 3. Results

### 3.1. Study Selection

**Figure 1:**
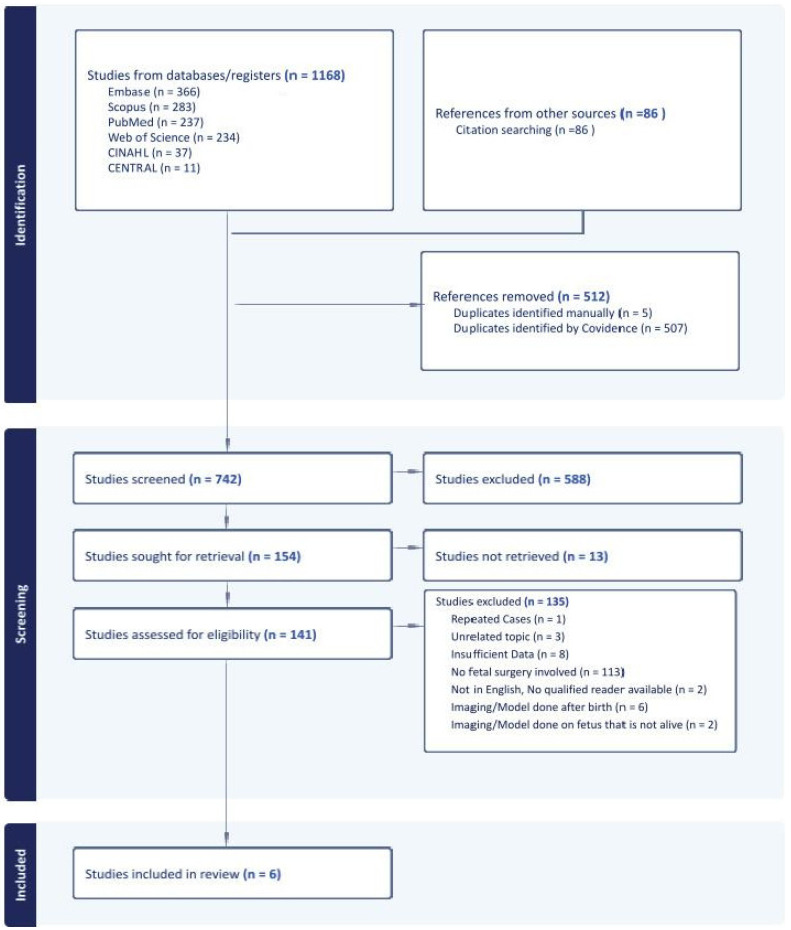
PRISMA flowchart.

The database search, along with a search of selected article citations, identified 1254 articles. Using automatic and manual comparison, 512 duplicates were removed, leaving 742 unique citations. After screening by title and abstract, 588 studies were excluded, with 154 remaining for full-text review. Upon reading the remaining citations, only six were found to meet the inclusion criteria and were included for data extraction. Studies were excluded upon full-text review for the following reasons: unrelated topic (3), insufficient data (8), full text not available (13), no fetal surgery involved (113), not in English with no qualified reader available (2), imaging/model performed after birth (6), repeated cases (1), and imaging/Model performed on a fetus that is not alive (2). Insufficient data were invoked when a study tangentially mentioned the use of printing in fetal surgery without providing case details or how the print was used. The full PRISMA flowchart can be seen in Figure 1 and characteristics of the extracted articles can be found in Table 1. Included studies used 3D prints for the surgical planning of spina bifida repair (2) or the determination of need and methodology for an EXIT procedure to secure the fetal airway (4).

This flowchart details the review process of articles from search acquisition to study inclusion. Studies not retrieved refers to those for which the full text was not available.

### 3.2. Characteristics of Included Studies

Characteristics of included studies This table graphically presents the various characteristics of the studies included in this scoping review (Table 1).

#### 3.2.1. Publication Timeline

The studies included in this scoping review were published between 2015 and 2022, showcasing a recent and evolving interest in the use of 3D printing for fetal surgery planning.

#### 3.2.2. Geographic Distribution

Although the majority of studies originated from the United States, indicating a primary focus within the U.S. healthcare context, one study conducted in Israel demonstrates that this technology is being developed simultaneously in different countries.

#### 3.2.3. Article Types

Diverse publication types were identified, including three full-text articles, one short communication, and two abstracts. This diversity in publication types underscores a lack of standardization on how to report these types of novel cases.

#### 3.2.4. Study Design and Size

The types of studies included in this scoping review vary, encompassing three case reports, two case series, and one clinical trial. The studies involved a range of delivery methods, with twelve cesarean sections, nine vaginal deliveries, and nine cases where the delivery method was not reported. Notably, three surgical cases were unsuccessful, with two delivered by emergency cesarean section and the third not reported [15]. Each study size ranged from 1 to 19, totaling 34 fetuses across the included studies.

#### 3.2.5. 3D Printing Techniques

The 3D printing techniques employed varied across the studies. Two studies did not provide information on the 3D printing technique used. However, among the identified techniques were fused deposition modeling (FDM), stereolithography, and a combination of FDM and stereolithography. In one instance, the technique was not explicitly stated, but material selection suggested a likely use of FDM.

The 3D printing materials used also varied by study. Among the articles, four did not specify the materials used, one used acrylonitrile butadiene styrene, and another employed a “semi-hard resin”.

In the exploration of 3D printing applications for fetal surgery planning, two distinctive focal points emerged: spinal surgery, specifically for spina bifida repair, and planning for EXIT (ex utero intrapartum treatment) procedures.

### 3.3. Described Benefits

#### 3.3.1. Open Spina Bifida

In the realm of fetal spinal surgery for spina bifida, the integration of 3D printing technology has demonstrated significant benefits. Handler et al.’s. [16] study utilized 3D models to predict the need for patches in myelomeningocele (MMC) repair and as a sterile template to prepare the patch early intraoperatively. Three-dimensional MRIs were acquired of each patient and used to create a 3D model. The accurate sizing of the MMC defect allowed surgeons to determine that only six of the patients would require a patch. Sizing the patch early in the procedure allowed the surgeons to complete the repair within their goal of 30 min in all but one case. The authors felt that this ability to size the patch early reduced operative time and potentially contributed to a decrease in premature delivery and intraoperative complications [16].

Miller et al. [15] further harnessed the power of 3D printing for team training in two-port open fetoscopic MMC repair. Using 3D ultrasound to scan a patient with MMC, a 3D printed model of the lower back was created. Additionally, silicon and other materials were combined with the model to create a representative surgical simulator. This allowed the team to rehearse the procedure until operative times for each step were consistent and to determine the optimal instrumentation and suture material. The training experience with models was critical to creating a cohesive operative team that minimized clinical errors. It allowed the team to better anticipate specific aspects and challenges of the procedures [15].

**Figure 2:**
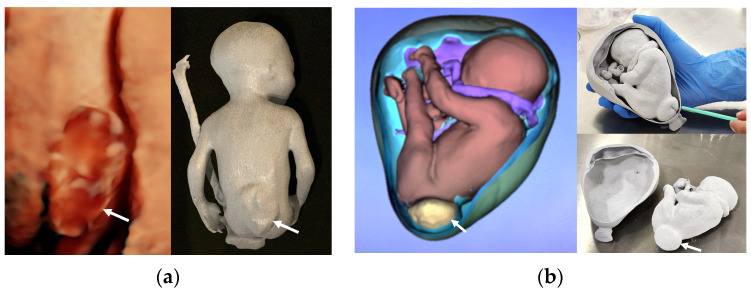
(**a**) Fetus with myelomeningocle and Chiari II malformation at 23 weeks’ gestation. Three-dimensional ultrasound and printed model built in a powder-based system showing myelomeningocele (arrow); (**b**) virtual and printed model (25 weeks) in powder system for medical training.

These studies collectively underscore the potential of 3D printing to refine surgical planning, improve procedural efficiency, and contribute to positive clinical outcomes in fetal spinal surgery for spina bifida (Figure 2).

#### 3.3.2. EXIT

**Figure 3:**
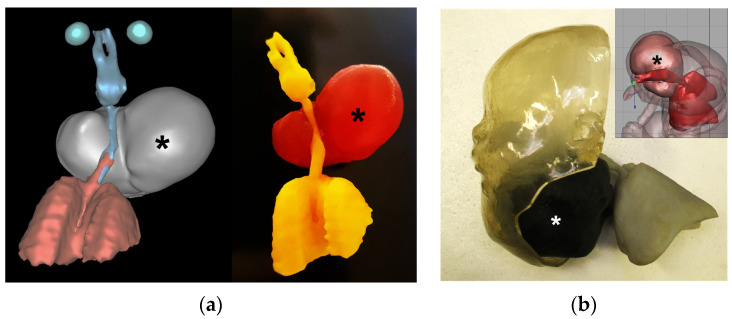
(**a**) Three-dimensional virtual and printed model showing cervical teratoma (*) at 37 gestational weeks. Note the patency of fetal airways; (**b**) 3D printed model built in photosensitive resin showing the tumor.

The utilization of 3D printing technology in planning for EXIT procedures has emerged as a valuable tool in addressing complex fetal anomalies and optimizing surgical strategies (Figure 3). Kelle et al.’s. [17] study used a full-fetal model created from MRI imaging. There was no explicit discussion of the benefits, only that it was used by an interdisciplinary team to prepare for surgery [17].

Shalev et al. [18] used 3D printing to avoid unnecessary attempts at intubation in order to decrease the time to gain airway control in a patient found to have a cervical multi-cystic lymphatic malformation. MRI images of the mandible, tongue, mass, larynx, and trachea were used to create a 3D model. Although the model was not used to create the initial plan for EXIT, it confirmed that EXIT was indicated and assisted in procedural planning. The model allowed the care team to identify relevant structures and determine which intubation method would be optimal. This enhanced planning provided the care team with a better understanding of the anatomy and pathology, allowing them to create a surgical plan with confidence [18].

Garcia de Paredes et al. [19] identified two fetuses, one with a cervical teratoma and one with an oropharyngeal teratoma that had the potential to complicate the fetuses’ airways and the physicians’ availability to establish a secure airway. In both cases, MRI was not able to portray the needed information. Both were imaged using 3D ultrasound to create 3D models of each fetus’s face and neck. Using the model, it was determined that fetus one would require an EXIT procedure. Furthermore, the model allowed the team to further characterize the fluid and solid components of the mass and facilitated their ability to successfully aspirate fluid from the mass before making an incision in the lower uterine segment. For fetus two, the model enabled the care team to determine that an EXIT procedure was not indicated. Overall, the authors felt the models allowed the care team to have more confidence in the anatomy and prepare for foreseen intraoperative interventions [19].

VanKoevering et al. [20] employed 3D printing to determine if an EXIT procedure was necessary for a patient with a protuberant bilateral cleft lip and palate with a prominent anteriorly displaced premaxilla and philtrum. During gestation, 2D imaging revealed a facial mass but visualization of the face was suboptimal due to fetal positioning and advanced gestational age. MRI imaging was used to create a 3D model of the craniofacial anatomy. Although the MRI did not provide new data, filtering and processing of the raw MRI data enabled improved visualization which showed that the airway was likely to be patent and that an EXIT procedure was not indicated. Furthermore, the hands-on nature of working with a printed model allowed the team to plan interventions like intubation—something they emphasized was not possible with digital models. Finally, the use of the model allowed the care team to have more confidence in their management plan [20].

Overall, these studies collectively underscore the utility of 3D printing in EXIT procedure planning, offering enhanced visualization, improved decision making, and increased confidence in surgical interventions.

### 3.4. Shortcomings

#### 3.4.1. Open Spina Bifida

Despite these promising benefits, certain shortcomings have been acknowledged in the literature. Miller et al. [15], in their programmatic study, noted longer surgery times, attributing them to significant limitations of the simulator in addressing the full range of surgical challenges that can arise during the procedure [15]. Handler et al. [16] did not explicitly discuss limitations, leaving a potential gap in the comprehensive understanding of the challenges associated with 3D printing in this context [16].

#### 3.4.2. EXIT

Challenges and limitations also persist in the utilization of 3D printing technology for EXIT procedure planning. Shalev et al. [18] noted general challenges associated with 3D modeling in utero, such as small tissue size and imaging limitations, the fetal position may not be in a standard anatomic plane, movement artifact, and that imaging with MRI uses larger slices and has poor tissue differentiation resulting in a poorer resolution model. Specific to their case, they were constrained by time to use the 3D printer available and therefore had to use a semi-hard material. Using an industrial printer, the tongue could have been printed in a flexible material to create a high-fidelity model that more accurately simulates the tissue characteristics [18].

As noted, fetal positioning can present a challenge by limiting the ability to fully image the fetus. The model used by Garcia de Paredes et al. [19] for fetus one was created from second-trimester imaging. The team attempted to create an updated third-trimester model but fetal positioning limited their ability to obtain adequate 3D ultrasound images. In addition, the authors felt that reporting guidelines for 3D printing in obstetrics and gynecology should be developed [19].

Kelle et al. [17] and VanKoevering et al. [20] did not provide an analysis of the limitations of the models they employed [17,20]. Future research should strive to address these limitations comprehensively, ensuring a thorough understanding of the practical considerations and technical challenges associated with the integration of 3D printing technology in fetal surgery planning.

## 4. Discussion

To our knowledge, this is the first scoping review to assess the current state of the use of 3D printed models for fetal surgery planning. Three-dimensional printing has been used for medical applications since the early 2000s starting with dental implants and custom prosthetics [1]. Since then, its use has been applied to the fields of neurosurgery, ophthalmology, orthopedics, plastic surgery, general surgery, oral and maxillofacial surgery, and others [2,21].

Other reviews have covered the use of 3D printing in obstetrics and gynecology [22]. For example, by printing molds that can be used to make the final product, a custom pessary was created for a 90-year-old woman [23]. A 3D printed vaginal mold was used to help create a neovagina in patients with vaginal agenesis who underwent vaginoplasty [24]. Three-dimensional printing has also been used to develop simulators for minimally invasive myomectomy [25], hemorrhagic cervical cancer [26], perineal repair [27], and pelvic examinations [28]. Uses in OBGYN for surgical planning include cesarean delivery in a patient with multiple myomas [29], complex myomectomy [30], and radical hysterectomy in a patient with cervical cancer [22,31]. However, no past reviews have focused exclusively and systematically on fetal surgery planning.

The primary benefit of 3D models is the ability to produce patient-specific models and case-specific medical equipment. Custom tools and implants can improve surgery times, patient recovery, and surgical outcomes. In addition, traditional manufacturing operates on the economy of scale, becoming less expensive as production increases. After the upfront cost of a printer, which can be expensive, the cost to print a 3D object can be minimal depending on the printed material. With time, printer costs have decreased, and the speed, resolution, and accuracy have increased. This means that highly complex products or those requiring frequent modifications can be produced without the high costs and long waiting times of traditional manufacturing [1].

As a well-established technology in the medical field, 3D printing is beginning to be applied in fetal surgery and is poised to provide advantages and opportunities that were previously impossible. Garcia de Paredes et al. [19], Shalev et al. [18], and VanKoevering et al. [20] demonstrated that printed models were instrumental in deciding whether an EXIT procedure was indicated. Increased confidence and the ability to improve visualization were key benefits. The described benefits were more varied for the spina bifida cases. Handler et al.’s. [16] model allowed them to standardize the operative time. However, Miller et al.’s. [15] simulator limitations contributed to increased surgical time. Their primary benefit was using the model to build team cohesion and minimize clinical errors. However, they all felt that the model helped to anticipate intraoperative challenges.

Three-dimensional printing has the potential to integrate itself into the foundation of fetal surgery. The ability to generate custom patient-specific models has proven its benefits. These benefits are further amplified when applied to a field in which there can be no physical examination of the patient. The ability to decrease surgical time can improve outcomes because premature labor and delivery are very serious complications of fetal surgeries. In addition, the teams involved in fetal surgeries often come from several different medical fields. The ability to use a patient-matched model to disseminate information and perform surgical rehearsals can help improve patient safety and team cohesion. The extracted studies demonstrate that 3D patient-specific models can provide important advantages to surgical teams, but a more robust set of literature is needed to determine how significant its advantages can be.

There are currently several different 3D printing systems on the market which are all based on manufacturing by layer deposition [32]. One of the most important features of 3D printing is the possibility of manufacturing parts with significant geometrical complexity, a process in which conventional technologies are lengthy and more expensive, affecting both the time taken to launch the product commercially and the total costs of production [32]. Three-dimensional printing technologies currently can be designated by the physical state of the materials to be transformed, for example, solid-based systems—associated with non-powder formats, such as sheets or thermoplastic extruded filaments, powder-based systems—associated with sintering or agglutination of grain particles, and liquid-based systems—associated with photopolymer resins [32].

Some studies omitted crucial details regarding the printing material, method, and specific printer utilized. Handler [16], Kelle [17], and VanKoevering [20] failed to discuss the limitations of their models, whereas Kelle et al. [17] also neglected to discuss the benefits of the models. Handler et al. [16] reported using their model as a sterile template but did not provide details on the sterilization method. The benefits and limitations that were described in the included studies were qualitative and lacked patient-matched controls. Additionally, no author discussed the rationale for their choice of imaging methods. Lastly, all studies, except one, were case reports or case series. These studies lack the rigor of controlled studies, which can limit their generalizability.

This scoping review provides valuable insights. A scoping review produces a summary and synthesis of the existing literature to determine gaps in science and specific areas. It provides opportunities to create new hypotheses and ideas for future research in the area [12]. However, it is also essential to acknowledge its limitations. Firstly, it excludes gray literature because it is not peer-reviewed [12]. The scoping review process, while valuable, has inherent limitations [12]. The first is publication bias, as studies with positive or significant results are more likely to be published. Additionally, the search strategy may not have captured all relevant studies, particularly those not indexed in the selected databases. Finally, the interpretation of the findings is subjective and influenced by the authors’ expertise and biases [12].

Additionally, we did not consider studies showing the proof of concept that imaging could be used to create a 3D model of a fetus because those studies did not apply the model in any way. For example, Tutschek et al. [33] used transvaginal ultrasound to produce early gestation fetal models [33]. Chen et al. [34] used 3D ultrasound to scan and print a 27-week fetus’ normal heart demonstrating that it was feasible on a moving structure without ionizing radiation or contrast media [34]. Guo et al. [35] created a 3D multicolor model of a 24-week fetus heart with tetralogy of Fallot [35]. Huang et al. [36] used 4D ultrasound print models of congenital heart disease including persistent truncus arteriosus, double inlet left ventricle, single atrium and ventricle, and transposition of great arteries [36]. Werner et al. [37] printed fetuses with encephalocele, sacrococcygeal teratoma, achondrogenesis, a fetal skeleton with femoral hypoplasia, and other conditions [37]. Jarvis et al. [38] used MRI to print fetal brains with ventriculomegaly, interhemispheric cysts, lissencephaly, and matched normal brains.

## 5. Conclusions

In conclusion, based on actual published studies in the literature, 3D printing models have been applied to plan for fetal surgeries, more specifically for EXIT procedures and fetoscopic surgical repair of spina bifida. The potential benefits of 3D printing in fetal surgery are enormous. Additional use of 3D printing for other types of fetal surgeries should still be explored.

## Figures and Tables

**Table 1:** Characteristics of included studies.

First Author	Title	Study Design	Maternal Age [Years]	Maternal Previous Gestation	Number of Patients	Gestational Age at Imaging [Weeks]	Gestational Age at Surgery [Weeks]	Diagnosis	What Was the Model Primarily Used for	Anatomical Region Modeled	3D Printing Material/Method	Imaging Method Used	Delivery Method/Age [Weeks]	Fetal Interventions	Described Benefits
Spina bifida															
Miller (2021) [15]	Implementation Process and Evolution of a Laparotomy-Assisted 2-Port Fetoscopic Spina Bifida Closure Program	Clinical Trial	31.1 ± 7.2	-	19	-	25.1 [22.9–25.9]	Open Spina Bifida (12 myelomeningocele, 7 myeloschisis)	Surgical Simulator	MMC Defect	Method- Not Specified Materials-Not Specified, Silicone skin covering	Ultrasound	9/16—vaginal 7/16—cesarean 2/3 unsuccessful cases delivered by emergency cesarean (did not report last case) Median delivery GA was 37 weeks (range 27–39.6)	Fetoscopic OSB closure	The model was felt to be critical in successfully building a cohesive operative team which minimized clinical errors Patient-matched models allowed specific aspects or challenges for each procedure to be better anticipated. Practice sessions allowed the team to improve surgical technique and efficiency
Handler (2016) [16]	Three-dimensional modeling of fetal myelomeningocele	Case Series	-	-	9	-	-	Myelomeningocele	Predict the need for a patch Template to create a patch	MMC Defect	Method- Not specified Material- Acrylonitrile butadiene styrene (ABS)	MRI	-	Myelomeningocele repair with or without patch placement	3D modeling allowed surgeons to know in advance if a patch would be required. In those that required a patch, early intraoperative patch production for MMC closure, allowed for reduced intraoperative times & complications
								EXIT							
Kelle (2017) [17]	Delivery and management of complete ectopia cordis: A multidisciplinary approach	Case report	25	-	1	-	36	Pentalogy of Cantrell and large thoracoabdominal defect	Surgical Planning	Full Body	-	MRI	Cesarean delivery at 36 weeks	EXIT procedure to secure airway	-
Shalev (2021) [18]	Utility of three-dimensional modeling of the fetal airway for ex utero intrapartum treatment	Case report	31	-	1	32	38	Cervical multi-cystic lymphatic malformation	Identify the tongue/glottis position and practice intubation	Mandible, tongue, mass, larynx, and trachea	Method- Stereolithography Material- Not Specified	MRI	Cesarean delivery at 38 weeks	EXIT procedure to secure airway	Clearer understanding of the anatomy and pathology. Increased confidence in surgical plan
Garcia de Paredes (2022) [19]	Antenatal Three-Dimensional Printing for Ex Utero Intrapartum Treatment Procedures	Case series	1. 32 2. 22	-	2	1. 25 4/7 2. 27 4/7	1. 37 1/7 2. No intervention	1. Cervical teratoma 2. Oropharyngeal teratoma	Determine the necessity for EXIT procedure	Face/neck	1. Fused deposition modeling; unknown material 2. Stereolithography; unknown material	Ultrasound	1. 37 1/7; cesarean delivery 2. 33 6/7; cesarean delivery	1. U/S guided needle aspiration, EXIT Procedure to secure airway 2. None	1. Asses the relationship of mass to airway 2. Model showed no airway obstruction so EXIT procedure was not indicated Increased team confidence in anatomy and foreseen intra-operative interventions
VanKoevering (2015) [20]	Antenatal Three-Dimensional Printing of Aberrant Facial Anatomy	Case report	22	G1P0	1	32	-	Protuberant bilateral cleft lip and palate with a prominent anteriorly displaced premaxilla and philtrum	Determine the necessity for EXIT procedure	Face	Fused deposition modeling and stereolithography Unknown material	MRI	Cesarean section	None	Improved visualization of maxillofacial anatomy Hands-on experience allowed the team to plan potential interventions Increased confidence in the anatomy and potential interventions necessary

## Data Availability

No new data were created or analyzed in this study. Data sharing is not applicable to this article.

## Appendix A. Database Search Strategies

Pubmed (United States National Library of Medicine1809–present)

Searched 8-12-23, 1809–present

237 Records Retrieved

(“Fetus”[MeSH Terms] OR “Fetal Diseases”[Mesh] OR “Fetal Therapies”[Mesh] OR “Embryo, Mammalian”[Mesh] OR “Embryonic Structures”[Mesh] OR embyro*[All Fields] OR fetal*[All Fields] OR fetoscop*[All Fields] OR fetus*[All Fields] OR foetal[All Fields] OR foetoscop*[All Fields] OR foetus*[All Fields] OR “in-utero*”[All Fields] OR inutero[All Fields] OR prenatal*[All Fields] OR “pre-natal”[All Fields]) AND (“printing, three dimensional”[MeSH Terms] OR stereolithograph*[All Fields] OR stereo-lithograph*[All Fields] OR additive-manufactur*[All Fields] OR (“print*”[All Fields] AND ((“three”[All Fields] AND “dimension*”[All Fields]) OR “3D”[All Fields] OR “3-D”[All Fields])))

Web Of Science (Clarivate)

Searched 8-12-23

234 Results

Databases: Web of Science Core Collection: Science Citation Index Expanded (1945–present), Social Sciences Citation Index (1956–present), Arts & Humanities Citation Index (1975–present), Conference Proceedings Citation Index-Science (1990–present), Conference Proceedings Citation Index- Social Science & Humanities (1990–present), Emerging Sources Citation Index (2015–present), KCI-Korean Journal Database (1980–present), ProQuest Dissertations & Theses Citation Index, SciELO Citation Index (2002–present)

(TS = (embyro* OR fetal* OR fetoscop* OR fetus* OR foetal OR foetoscop* OR foetus* OR in-utero* OR inutero OR prenatal* OR pre-natal*)) AND TS = (stereolithograph* OR stereo-lithograph* OR additive-manufactur* OR (print* AND ((three AND dimension*) OR 3D OR 3-D)))

Scopus (Elsevier)

Searched 8-12-23, 1823–present

283 records retrieved

TITLE-ABS-KEY ((stereolithograph* OR stereo-lithograph* OR additive-manufactur* OR (print* AND ((three AND dimension*) OR (3d OR 3-d)))) AND (embyro* OR fetal* OR fetoscop* OR fetus* OR foetal OR foetoscop* OR foetus* OR in-utero* OR inutero OR prenatal* OR pre-natal))

Embase (Elsevier, Embase.com)

Searched 8-12-23, 1947–present

366 records retrieved

#3: #1 AND #2

#2: ‘fetus’/exp OR ‘fetus (anatomy)’/exp OR ‘fetus disease’/exp OR ‘fetal therapy’/exp OR ‘embryo’/exp OR embyro*:ti,ab,kw OR fetal*:ti,ab,kw OR fetoscop*:ti,ab,kw OR fetus*:ti,ab,kw OR foetal:ti,ab,kw OR foetoscop*:ti,ab,kw OR foetus*:ti,ab,kw OR ‘in utero*’:ti,ab,kw OR inutero:ti,ab,kw OR prenatal*:ti,ab,kw OR ‘pre natal’:ti,ab,kw

#1: ‘three dimensional printing’/exp OR stereolithograph*:ti,ab,kw OR ‘stereo lithograph*’:ti,ab,kw OR ‘additive manufactur*’:ti,ab,kw OR (print*:ti,ab,kw AND (three:ti,ab,kw AND dimension*:ti,ab,kw OR 3d:ti,ab,kw OR ‘3 d’:ti,ab,kw))

Cochrane Central Register of Controlled Trials: CENTRAL (Cochrane Library, Wiley)

Searched 8-13-23, from inception to 2023, Issue 8

11 records retrieved

([mh Fetus] OR [mh “Fetal Diseases”] OR [mh “Fetal Therapies”] OR [mh “Embryo, Mammalian”] OR [mh “Embryonic Structures”] OR embyro*:ti,ab OR fetal*:ti,ab OR fetoscop*:ti,ab OR fetus*:ti,ab OR foetal:ti,ab OR foetoscop*:ti,ab OR foetus*:ti,ab OR (in NEXT utero*:ti,ab) OR inutero:ti,ab OR prenatal*:ti,ab OR (pre NEXT natal*):ti,ab)

AND

([mh “printing, three dimensional”] OR stereolithograph*:ti,ab OR (stereo NEXT lithograph*:ti,ab) OR (additive NEXT manufactur*:ti,ab) OR (print*:ti,ab AND ((three:ti,ab AND dimension*:ti,ab) OR 3D:ti,ab OR “3 D”:ti,ab)))

CINAHL Plus with Full Text (Ebsco)

Searched 8-12-23, 1937–present

37 records retrieved

((MH “Fetus+”) OR (MH “Fetal Surgery”) OR (MH “Fetal Abnormalities”) OR (MH “Fetal Diseases+”) OR (MH “Embryo+”) OR (embyro* OR fetal* OR fetoscop* OR fetus* OR foetal OR foetoscop* OR foetus* OR in-utero* OR inutero OR prenatal* OR pre-natal))

AND

((MH “Printing, Three-Dimensional”) OR (stereolithograph* OR stereo-lithograph* OR additive-manufactur* OR (print* AND ((three AND dimension*) OR 3D OR 3-D))))
